# The additive value of platelet-rich plasma to topical Minoxidil in the treatment of androgenetic alopecia: A systematic review and meta-analysis

**DOI:** 10.1371/journal.pone.0308986

**Published:** 2024-08-28

**Authors:** Jia Yao, Lu Zhu, Meier Pan, Liling Shen, Yanli Tang, Liping Fan

**Affiliations:** Department of Dermatology, Zhejiang Provincial Dermatology Hospital, Huzhou, Zhejiang, China; Yuan Ze University, TAIWAN

## Abstract

**Objective:**

It still needs to be determined if platelet-rich plasma (PRP) has any added advantage over Minoxidil in treating androgenetic alopecia. We reviewed randomized controlled trials (RCTs) comparing scalp injections of PRP plus Minoxidil vs Minoxidil alone for managing androgenetic alopecia.

**Methods:**

All RCTs published on Embase, Cochrane Library, and PubMed comparing PRP plus Minoxidil vs. Minoxidil alone were eligible. The literature search was completed on 5 March 2024. The review was registered on PROSPERO (CRD42024509826).

**Results:**

Of five included RCTs, three had a high risk of bias, while one had some concerns. A systematic review of the studies showed that all trials reported better outcomes with PRP plus Minoxidil than with Minoxidil alone. Meta-analysis showed that hair density at one month (MD: 11.07 95% CI: 1.20, 20.94 I^2^ = 0%), three months (MD: 21.81 95% CI: 10.64, 33.00 I^2^ = 57%) and 5/6 months (MD: 17.80 95% CI: 7.91, 27.69 I^2^ = 80%) of follow-up was significantly better in the PRP plus Minoxidil vs the Minoxidil alone group. Meta-analysis of adverse events showed that the risk of adverse events was comparable in both groups (OR: 0.55 95% CI: 0.22, 1.36 I^2^ = 0%). The certainty of evidence on the GRADE assessment was "low to very low."

**Conclusion:**

Very low-quality evidence shows that the addition of injectable PRP to topical Minoxidil may improve outcomes in patients with androgenetic alopecia. The addition of PRP was found to improve hair density and patient satisfaction significantly. However, the small number of studies with a high risk of bias and heterogeneity in PRP preparation methods are significant limitations of current evidence. Further studies with larger sample sizes and uniform PRP preparation protocols are needed.

## Introduction

Androgenetic alopecia is a common hereditary condition resulting in hair loss. It affects about half of the population, leading to progressive loss of terminal hair on the scalp post-puberty. Androgenetic alopecia follows a characteristic distribution in both genders: males are losing hair in the vertex and frontotemporal regions, while females show diffuse hair loss at the crown and top of the head [1, 2]. The disease has an apparent genetic predisposition and is caused by excessive response to androgen. The hair follicles are sensitive to dihydrotestosterone, which shortens the anagen phase of the normal hair growth cycle, resulting in gradual thinning and shortening of hair follicles, which eventually disappear [3]. Androgenetic alopecia not only affects the appearance of the individual but also has a significant psychological impact, often leading to depression [4].

Finasteride and Minoxidil are among the Food and Drug Administration (FDA)-approved treatments for androgenetic alopecia [1]. Finasteride is a 5 alpha-reductase type 2 inhibitor administered orally and deemed efficacious in managing the disease. However, it leads to significant sexual side effects, limiting its general use [5]. Topical Minoxidil has gained widespread acceptance for managing androgenetic alopecia due to its ease of use, good results, and minimal local side effects. However, studies show that combining Minoxidil with add-on treatments may improve hair density more efficiently [5, 6].

One such treatment is based on using platelet-rich plasma (PRP), which has shown good potential for managing the disease. PRP is obtained by centrifugation of the patient’s venous blood to produce a concentrate of platelets. While the normal platelet count in humans is 1.5–4.5 × 10^5^/ml, the count in PRP is about 4-5x higher than in normal plasma [7]. The abundance of growth factors in PRP makes it a viable therapeutic modality in regenerative medicine [8]. It was demonstrated that PRP is efficacious in the treatment of androgenetic alopecia. A meta-analysis of 11 randomized controlled trials (RCT) has shown that PRP injections significantly increase the number of hair follicles, hair thickness, and density compared to placebo interventions [9]. A systematic review also shows that PRP has comparable efficacy to Minoxidil without major side effects [10]. However, the efficiency of PRP combined with Minoxidil in treating androgenetic alopecia is still unclear. Several studies [11–13] have been conducted to verify the added value of PRP in androgenetic alopecia, but no comprehensive review has been conducted to date. We report the results of the first systematic review and meta-analysis of RCT examining the efficacy of PRP with Minoxidil vs. Minoxidil alone for treating androgenetic alopecia.

## Material and methods

### Search methodology

The reporting of this review is following PRISMA guidelines [14] (S1 Table). We prospectively registered the protocol on PROSPERO (CRD42024509826). Embase, Cochrane Library, and PubMed’s publication repositories were systematically searched until 5 March 2024 for trials evaluating the additive effect of PRP with Minoxidil for alopecia. The search was supplemented by screening of clinicaltrials.gov for any ongoing studies. Literature was explored using the keywords: ‘androgenetic alopecia’, ‘male pattern baldness’, ‘female pattern baldness’, ‘hair loss’, ‘platelet-rich plasma’, ‘platelet-rich fibrin’, ‘PRP’, ‘PRF,’ and ‘minoxidil’. The complete list of search queries is appended as S2 Table. The entire process of searching and selecting studies was conducted by the two reviewers with the aid of an experienced medical librarian.

The search results of all databases were combined and then deduplicated using the software EndNote. The two investigators examined the title and abstract of each remaining study to assess primary eligibility. Irrelevant articles were eliminated at this stage. Potentially eligible studies were downloaded for further analysis. The reviewers carefully examined the full texts and included studies that met all inclusion criteria. The literature search was further supplemented by reading the bibliography of included studies for additional literature. Any discrepancies in the study selection were resolved in consultation with a third reviewer.

### Eligibility criteria

Criteria for inclusion were derived based on the PICOS methodology, where *the population* was cases of androgenetic alopecia irrespective of gender and grade. *The intervention* was PRP with topical Minoxidil. *The comparison* group was topical Minoxidil alone. Outcomes to be assessed were hair density and hair thickness noted on trichoscopy or photography. *The* included study types were RCTs only.

The exclusion criteria were:

1. Non-randomized studies and retrospective studies.
2. Unpublished data, thesis, and literature reviews.
3. Not reporting desired outcomes.
4. Duplicate studies. In the case of multiple publications from the same institute, the article with a larger sample size and longer follow-up was eligible for inclusion.

### Data management

Baseline details, including author information, study type, location, details of intervention and control group including the timing of injections, method of PRP preparation, dosage of Minoxidil, age and gender of participants, assessment methods, outcomes, and follow-up, were extracted from the studies by two reviewers (JY and LZ) on 15 March 2024. Disagreements were resolved after discussion with the third reviewer. Details were cross-checked for correctness. For missing data, the corresponding author was contacted by email once. If no response was received, we proceeded with the meta-analysis with the remaining studies. All data was extracted from studies confirmed to be included in the meta-analysis. The aim was to conduct a detailed qualitative analysis of outcomes and a quantitative analysis of hair density since it was the most reported outcome variable.

Two reviewers used the Cochrane Collaboration risk of bias-2 tool [15] to examine the quality of studies. Studies were judged for the randomization process, deviation from intended intervention, missing outcome data, measurement of outcomes, selection of reported results, and overall risk of bias. Disagreements were resolved after discussion with the third reviewer. We examined the certainty of evidence of all outcomes using the GRADE (Grading of Recommendations, Assessment, Development, and Evaluations) assessment tool (https://gdt.gradepro.org/app/).

### Statistical analysis

A random-effects meta-analysis was conducted for available data on Review Manager 5.3 (Cochrane Collaboration, 2014). Data on hair density at different follow-up time points was pooled to calculate mean difference (MD) with 95% confidence intervals (CI). Adverse events were combined using odds ratio (OR). Forrest plots were produced to present the results. The software measured the heterogeneity among studies using the I value and the Chi-square test. P< 0.05 was considered statistically significant, and an I^2^ value of 50% or more indicated the presence of heterogeneity [16]. As too few studies were available for quantitative analysis, sensitivity analysis was not conducted, and a funnel plot was not drawn.

## Results

### Characteristics of included studies

**Fig 1:**
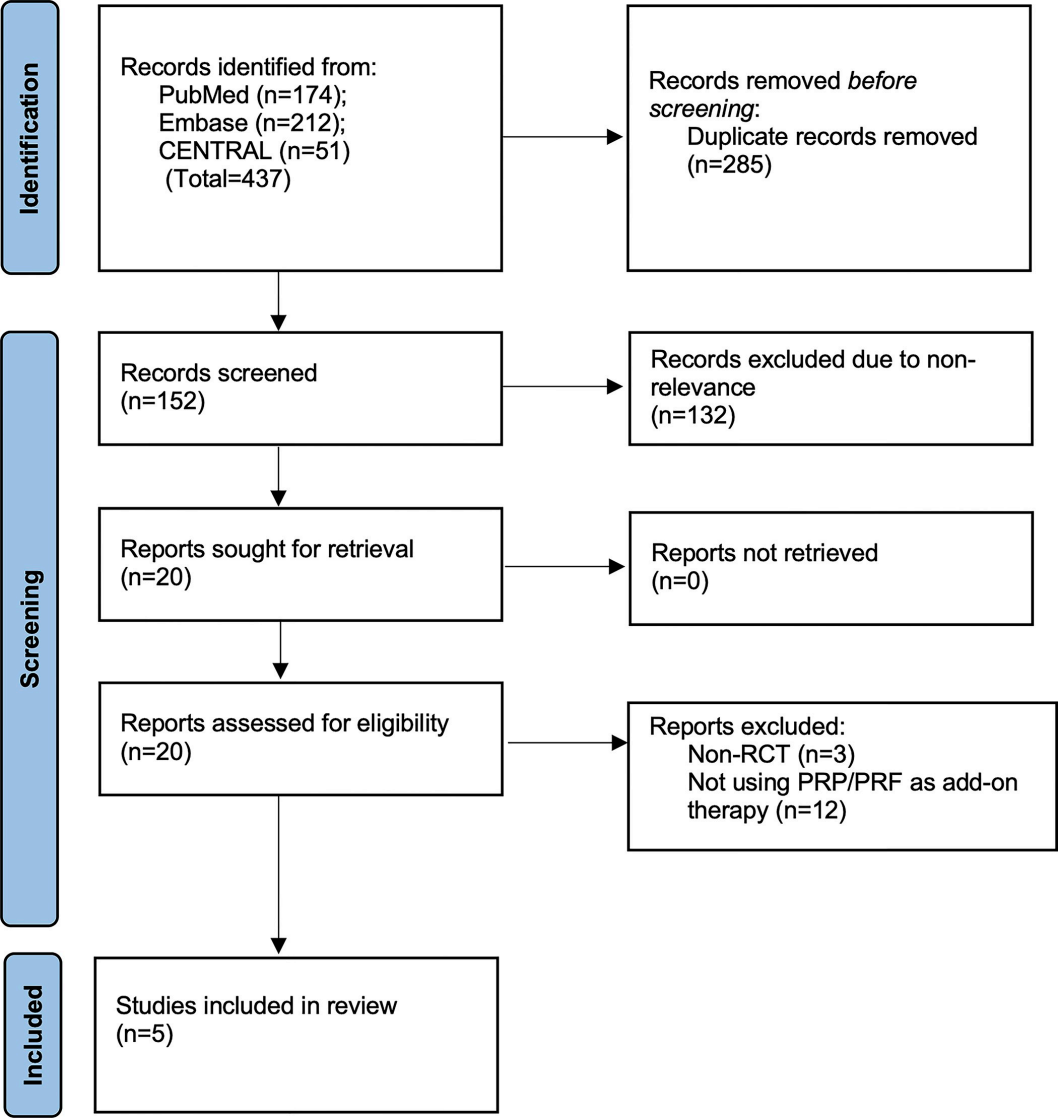
PRISMA flowchart of the review.

Stepwise search results are presented in the PRISMA diagram (Fig 1).

The reviewers retrieved 437 articles from Embase, Cochrane Library, and PubMed databases. No additional RCT was found on clinicaltrials.gov. The deduplication process eliminated 285 studies following preliminary screening, and 152 studies underwent complete text analysis. Finally, five studies [11–13, 17, 18] met the inclusion criteria. A bibliography search also did not yield any new articles. List of excluded studies with reasons is appended as S3 Table.

Qualitative data extracted from the five RCTs is shown in Table 1. All RCTs were conducted between 2020 and 2023 and originated from India, China, and Egypt. One study used platelet-rich fibrin (PRF), while the remaining trials used PRP. Two trials used the single spin method to prepare PRP, while others used the double spin technique. All studies injected PRP at monthly intervals in the scalp. The number of injections ranged from three to six. All patients received topical Minoxidil 5%. The sample sizes of the studies were not high and ranged from 20 to 50 in each group. Trichoscopy and photographic evaluation were conducted at different follow-up intervals to assess the effect of interventions. Follow-up ranged from five to nine months.

**Table 1:** Details of included studies.

Study	Location	Study type	Intervention	Control	Centrifugation protocol	Groups	Sample size	Age (years)	Males	Assessment method	Follow-up
Wu 2023 [18]	China	RCT	Three PRP treatment sessions at 1-month intervals with topical Minoxidil 5%. Injected at a dose of 0.05–0.1 mL/ cm^2^. Basic fibroblast growth factor 20 μg/ 1ml of PRP was added.	Topical Minoxidil 5% twice daily	1^st^ spin: 3300 rpm for 4 min 2^nd^ spin: 3200 rpm for 3 min	Intervention Control	25 25	39.2 37	12 16	Trichoscopy and global photographs	6 months
Pachar 2022 [17]	India	RCT	Six PRP treatment sessions at 1-month intervals with topical Minoxidil 5%.	Topical Minoxidil 5% twice daily	1^st^ spin: 1200 rpm for 8 min 2^nd^ spin: 2400 rpm for 4 min	Intervention Control	50 50	NR	50 50	Trichoscopy and global photographs	6 months
Gowda 2021 [11]	India	RCT	Four PRP treatment sessions at 1-month intervals with topical Minoxidil 5%.	Topical Minoxidil 5% twice daily	3600 rpm for 5 min (single spin)	Intervention Control	30 30	NR	30 30	Trichoscopy and global photographs	5 months
Ramadan 2021 [13]	Egypt	RCT	Three to six PRP treatment sessions at 1-month intervals with topical Minoxidil 5%.	Topical Minoxidil 5% twice daily for males and once daily for females	Single spin for 10 mins	Intervention Control	42 42	NR	NR	Trichoscopy and global photographs	9 months
Singh 2020 [12]	India	RCT	Three PRP treatment sessions at 1-month intervals with topical Minoxidil 5%.	Topical Minoxidil 5% twice daily	1^st^ spin: 2200 rpm for 12 min 2^nd^ spin: 3000 rpm for 6 min	Intervention Control	20 20	25.6 26.9	20 20	Trichoscopy and global photographs	5 months

RCT, randomized controlled trial; NR, not reported; PRF, platelet-rich fibrin; PRP, platelet-rich plasma.

### Risk of bias

Table 2 presents the quality analysis of the studies. Only one RCT [18] was of high quality with a low risk of bias. None of the other studies [11–13, 17] reported the randomization method and allocation concealment in detail. Two trials [11, 17] were unblinded, while one [12] did not report the method of blinding.

**Table 2:** Risk of bias analysis.

Study	Randomization process	Deviation from intended intervention	Missing outcome data	Measurement of outcomes	Selection of reported result	Overall risk of bias
Wu 2023 [18]	Low risk	Low risk	Low risk	Low risk	Low risk	Low risk
Pachar 2022 [17]	Some concerns	Low risk	Low risk	High risk	Low risk	High risk
Gowda 2021 [11]	Some concerns	Low risk	Low risk	High risk	Low risk	High risk
Ramadan 2021 [13]	Some concerns	Low risk	Low risk	Low risk	Low risk	Some concerns
Singh 2020 [12]	Some concerns	Low risk	Low risk	Some concerns	Low risk	High risk

### Qualitative analysis

In their RCT, Wu et al. [18] measured hair density, thickness, vellus hair, terminal hair, vellus hair ratio, telogen hair ratio, and hair growth rate. The authors noted that hair density, hair thickness, terminal hair, telogen hair ratio, and hair growth rate were significantly increased with the addition of PRF to topical Minoxidil as compared to Minoxidil alone. However, the two groups had no difference in vellus hair and vellus hair ratio. The patient satisfaction was also better in the PRF plus Minoxidil group than in the Minoxidil alone group.

The RCT by Pachar et al. [17] examined only hair density, and noted significantly increased hair density in the PRP plus Minoxidil group compared to Minoxidil alone. Patients’ perception of improvement was better in the combined group. The improvement in the Dermatology Life Quality Index was also significantly better in the PRP plus Minoxidil group compared to Minoxidil alone.

Ramadan et al. [13] evaluated improvement in hair density and diameter between the intervention and control groups in their RCT. Since the study did not report baseline and final hair density values, the trial could not be included in the meta-analysis. The authors noted significant hair density and diameter improvement by adding PRP to Minoxidil. Patients in both groups were satisfied with the treatment.

The RCT of Gowda et al. [11] evaluated the difference in pre- and post-treatment hair growth on a 5-point scale as the primary analysis. A secondary analysis consisted of a change in global photographic assessment for hair growth on a 7-point scale. The authors noted significantly better results in both the primary and secondary analysis for the PRP plus Minoxidil group compared to the Minoxidil group.

Singh et al. [12] also evaluated hair density in their RCT. The authors noted significant improvement in hair density in the PRP plus Minoxidil group compared to the Minoxidil group at all follow-up periods. Patient satisfaction was also significantly higher in the combined group than in Minoxidil alone.

### Quantitative analysis

**Fig 2:**
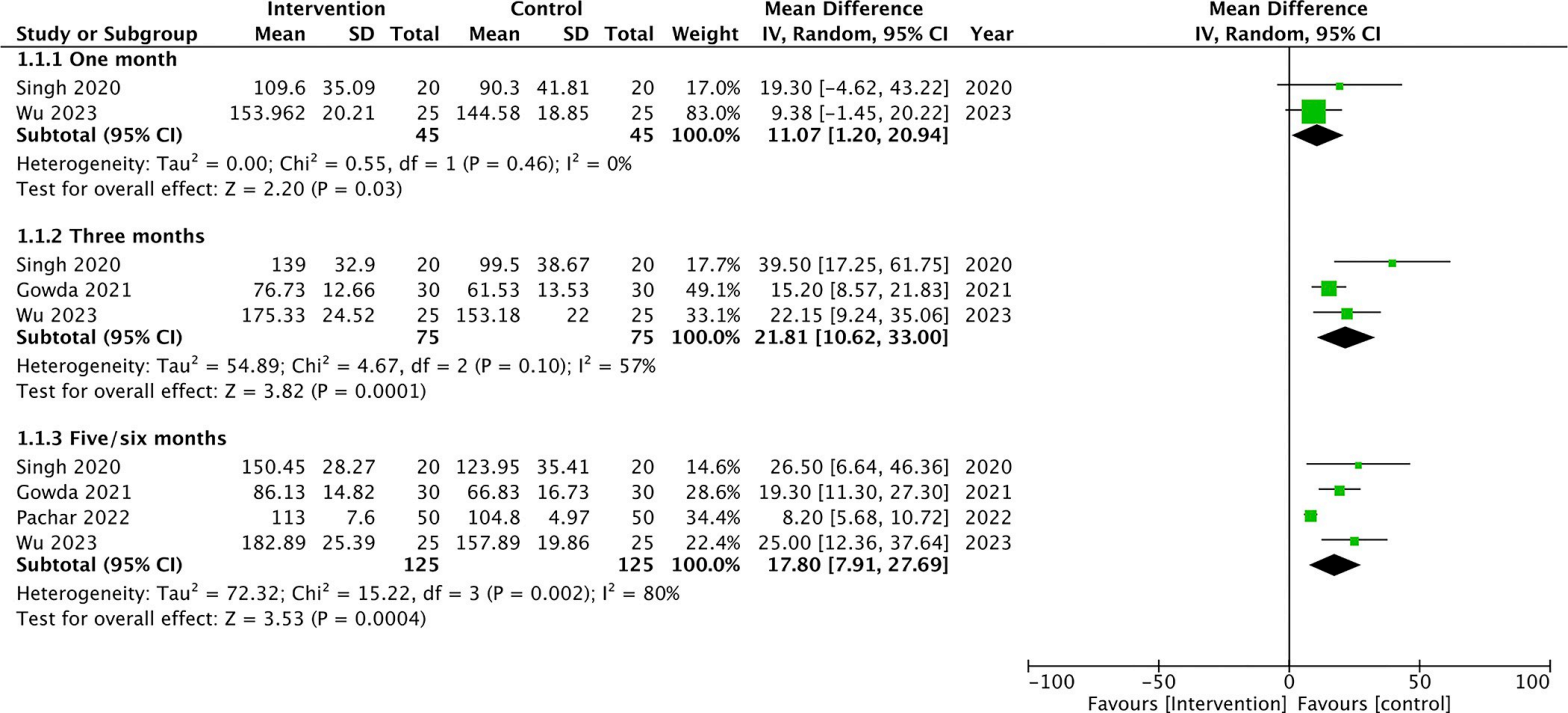
Meta-analysis of hair density between PRP plus Minoxidil vs minoxidil groups.

Quantitative data extracted from studies is provided in S4 Table. A meta-analysis could be conducted only for hair density, as the included studies did not uniformly report other efficiency parameters. Further, we compared the final scores between the two groups and not the change in scores. Meta-analysis showed that hair density was significantly improved in the PRP plus Minoxidil group as compared to Minoxidil alone at one month (MD: 11.07 95% CI: 1.20, 20.94 I^2^ = 0%), three months (MD: 21.81 95% CI: 10.64, 33.00 I^2^ = 57%) and 5/6 months (MD: 17.80 95% CI: 7.91, 27.69 I^2^ = 80%) of follow-up (Fig 2). GRADE assessment showed that certainty of the evidence was low for one-month results and very low for 3-month and 5/6-month results (S5 Table).

**Fig 3:**
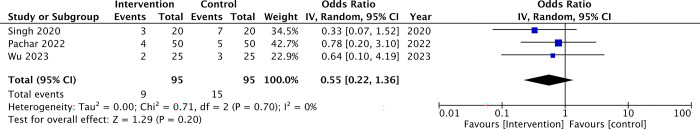
Meta-analysis of adverse events between PRP plus Minoxidil vs Minoxidil.

A meta-analysis of adverse events showed no difference in the risk of adverse events between the intervention and control groups (OR: 0.55 95% CI: 0.22, 1.36 I^2^ = 0%) (Fig 3). GRADE assessment showed that the certainty of evidence was very low (S5 Table). Table 3 shows the types of adverse events reported by the studies.

**Table 3:** Adverse events reported by the included studies.

Study	PRP + Minoxidil group	Minoxidil group
Wu 2023 [18]	Minimal pain, redness, and pinpoint bleeding	Itchy scalp
Pachar 2022 [17]	Erythema, burning, fever, persistent pain, edema, and flaking*	
Gowda 2021 [11]	Pain	Pruritis, seborrheic dermatitis
Ramadan 2021 [13]	Pain, headache, scalp irritation, increase in facial hair	Scalp irritation, increase in facial hair
Singh 2020 [12]	Pain, pruritis, increase in facial hair	Pain, pruritis, increase in facial hair, hyperpigmentation

*Types of adverse events reported for both groups combined

## Discussion

Out of the numerous therapeutic modalities available for androgenetic alopecia, topical Minoxidil is one of the few FDA-approved treatments. It was initially approved in 1988 as the first-line treatment for mild‐to‐moderate alopecia in men [19]. The discovery of hypertrichosis as a side-effect of oral Minoxidil in the 1960s led to the development of topical formulations for treating androgenetic alopecia [20]. Since then, 2% and 5% of Minoxidil foam and liquid solutions have been widely used with varying clinical efficacy [21]. Minoxidil has the most beneficial effect on the vertex and frontal regions, where it diminishes hair loss by increasing the anagen phase. It also stimulates hair regrowth with increased hair diameter and density. While the exact mechanism of Minoxidil in increasing hair growth remains elusive, it is postulated that Minoxidil increases vascular endothelial growth factor expression, which increases dermal papilla vascularization, especially around the anagen hair follicle [22]. Also, Minoxidil is converted into its active form of minoxidil sulfate by sulfotransferase present in the outer root sheath, by which the agent maintains its effect on the hair follicles rather than the surrounding skin. The active form binds to the adenosine triphosphate-sensitive potassium channels and relaxes the surrounding smooth muscle, promoting cutaneous blood flow and hair growth [23]. Numerous trials have demonstrated the efficacy of Minoxidil topical and oral formulations for androgenetic alopecia [24]. A meta-analysis by Gupta et al. [24] have shown that total hair count at 24 weeks was significantly better with oral Minoxidil as compared to topical formulations. However, off-label use of oral Minoxidil for alopecia leads to significant side effects like postural hypotension, fluid retention, and facial hypertrichosis. Topical formulations require frequent use and if the patient discontinues treatment progressive hair loss begins [1].

Numerous combination therapies have been recommended to overcome topical Minoxidil’s limitations and improve treatment efficacy. Chen et al. [5] A meta-analysis of five RCTs has shown that Finasteride added to topical Minoxidil has better therapeutic efficacy and similar safety as compared to Minoxidil alone. Another review by Abdi et al. [6] found that microneedling with topical Minoxidil results in better hair growth in patients with androgenetic alopecia than in Minoxidil alone. A recent network meta-analysis by Gupta et al. [25] that included 27 RCTs compared various combination therapies and found that microneedling with Minoxidil resulted in the highest hair growth compared to other combinations of PRP, microneedling, and Minoxidil. However, just one trial evaluating the combination of PRP and Minoxidil was included in their review, significantly limiting the conclusions.

Our review, for the first time, presents evidence of the additive value of PRP to topical Minoxidil for treating androgenetic alopecia. We synthesized data from five recently published RCTs to show that combining PRP and Minoxidil may result in better hair density than Minoxidil monotherapy. Further, a detailed qualitative synthesis of studies also demonstrated better overall outcomes and improved patient satisfaction with combination therapy. The results of the current review resonate with the outcomes of previous studies demonstrating the efficacy of PRP for androgenetic alopecia [7, 26]. Prior systematic reviews of RCTs have shown that PRP increases hair density and thickness in patients with androgenetic alopecia [7, 26].

Nevertheless, our results should be interpreted with caution as there was just one trial with a low risk of bias in the analysis. All other trials had a high risk of bias or some concerns that may limit the credibility of the results. GRADE assessment of evidence showed that the certainty of evidence was low to very low. Furthermore, we could assess outcomes only up to six months as no long-term data was available from the included trials. Given this fact, the current evidence must be supplemented by further high-quality trials to provide better-quality evidence.

Another critical point to consider is whether the statistically significant difference between the intervention and control groups translates into clinically relevant results for the patient. While we noted that the MD was statistically significant at all follow-up times, the difference between the two groups was just 11 to 21 hairs. Clinical relevance of outcomes can be gauged by the concept of "Minimal Clinically Important Difference" (MCID), which is the slightest change in the outcome perceived by the patient as beneficial and which mandates a change in healthcare protocols [27]. Unfortunately, there is no validated data on the MCID for hair density. Only further research can elucidate if adding PRP to Minoxidil results in clinically relevant outcomes.

In terms of adverse events, the meta-analysis did not find any difference between the intervention and control groups, albeit the number of studies was too small. In general, PRP does not have any significant side effects as it is derived from autologous blood. Injection site pain, temporary pain and tenderness, itching, headache, and mild bleeding may be noted after the injection [26]. None of the included trials reported any major adverse events of PRP injections.

The efficacy of PRP is attributable to the high concentration of growth factors. PRP is prepared from the patient’s blood, which is centrifuged to obtain a three- to eight-fold higher platelet concentration. The alpha-granules of concentrated platelets release numerous growth factors like the vascular endothelial growth factor, fibroblast growth factor, platelet-derived growth factor, epidermal growth factor, and insulin-like growth factor [28]. These act on the dermal papilla cells and cause an increase in phosphorylated extracellular signal-regulated kinases and protein kinase B, improving cell growth and survival and reducing apoptosis. Further, PRP also increases the concentration of antiapoptotic protein Bcl-2, which controls the expression of apoptotic molecules and increases cell survival [28]. An important caveat in the efficacy of PRP is its preparation method. In our review, the studies differed in the preparation methods, with some using the single spin method while others using the double spin method. The centrifugation speed and time also differed between trials. In this context, the Indian Association of Dermatologists, Venereologists, and Leprologists has provided recommendations on the optimal method of PRP preparation for dermatological use. They suggest using the double-spin manual method with 100–300 g for 5–10 min for the first spin and 400–700 g for 10–17 min for the second spin. The recommended platelet concentration in PRP is 1–1.5 million platelets/μL [29]. Future studies should use standardized PRP preparation techniques to provide better-quality evidence.

Ehrenfest et al. [8] have classified PRP into four types based on the presence of leucocytes and fibrin. Broadly, they are PRP and platelet-rich fibrin (PRF) with or without leucocytes. At this point, it is unclear which has better efficacy for alopecia. Further, none of the studies mentioned the type of PRP used. The use of PRF has been recently reported for alopecia [30]. PRP and PRF are generated from autologous blood but differ in the preparation methods. PRP is collected in anticoagulant vials, while PRF is without any anticoagulant, often forming a fibrin-rich clot. Injectable formulations of PRF have also been developed, which are prepared by low-speed centrifugation of blood in non-glassed centrifugation tubes [31]. The injectable PRF has the advantage of a higher volume of growth factors, which are released over a prolonged duration [32]. To date, no RCTs have been conducted on the utility of PRF for androgenetic alopecia. Given the efficacy of PRP, it would be interesting to study if PRF results in further better outcomes in cases of alopecia.

There are limitations to our review. Firstly, only five RCTs were available in the literature. The sample size of many of the included studies was not high. Secondly, there was substantial variability in the PRP preparation protocols, with studies varying in the numbers of spins and centrifugation time, which could alter the concentration of platelets. The ideal platelet concentration in PRP has been identified as 1.5 × 10^6^/μL; higher or lower concentrations may alter the growth potential [4]. The number of PRP injections and severity of the disease also varied between studies, possibly contributing to interstudy heterogeneity in the meta-analysis. However, a subgroup analysis was impossible due to a low number of studies. Thirdly, outcomes were not uniformly reported by the included studies. We could perform a meta-analysis for only hair density as outcomes like hair growth, hair diameter, and patient satisfaction were either not reported or examined by different scales. Fourthly, most studies did not report the baseline grade of androgenetic alopecia in the intervention and control groups. We, therefore, could not examine the efficacy of the treatments in different grades of the disease. Fifthly, data was available for a maximum follow-up of 5/6 months in the included studies. There is no long-term data on the effectiveness of adding PRP to Minoxidil. Lastly, there was only one high-quality RCT [18], while all others had a significant bias in the randomization method and blinding of outcome assessment.

### Conclusions

Very low-quality evidence shows that the addition of injectable PRP to topical Minoxidil may improve outcomes in patients with androgenetic alopecia. The addition of PRP was found to improve hair density and patient satisfaction significantly. However, the small number of studies with a high risk of bias and heterogeneity in PRP preparation methods are major limitations of current evidence. Further studies with larger sample sizes and uniform PRP preparation protocols are needed.

## Supporting information

S1 Table PRISMA checklist. (DOCX)

S2 Table Search strategy. (DOCX)

S3 Table List of excluded studies with reasons. (DOCX)

S4 Table Data extracted from studies. (DOCX)

S5 Table GRADE assessment of evidence. (DOCX)
