# Supplementary material for: The additive value of platelet-rich plasma to topical Minoxidil in the treatment of androgenetic alopecia: A systematic review and meta-analysis
Source: PLoS One. 2024 Aug 28;19(8):e0308986. doi: 10.1371/journal.pone.0308986 (PMC11356437; doi:10.1371/journal.pone.0308986)
Supplement: S2 Table — (DOCX) [file pone.0308986.s002.docx]

Supplementary Table 2: Search strategy

| **Query** | **Search Details** |
| --- | --- |
| (((((androgenic alopecia) OR (male pattern baldness)) OR (female pattern baldness)) OR (hair loss)) AND (PRP)) AND (minoxidil)) | ("alopecia"[MeSH Terms] OR "alopecia"[All Fields] OR ("androgenic"[All Fields] AND "alopecia"[All Fields]) OR "androgenic alopecia"[All Fields] OR ("alopecia"[MeSH Terms] OR "alopecia"[All Fields] OR ("male"[All Fields] AND "pattern"[All Fields] AND "baldness"[All Fields]) OR "male pattern baldness"[All Fields]) OR ("alopecia"[MeSH Terms] OR "alopecia"[All Fields] OR ("female"[All Fields] AND "pattern"[All Fields] AND "baldness"[All Fields]) OR "female pattern baldness"[All Fields]) OR ("alopecia"[MeSH Terms] OR "alopecia"[All Fields] OR ("hair"[All Fields] AND "loss"[All Fields]) OR "hair loss"[All Fields])) AND ("pharmacol res perspect"[Journal] OR "prp"[All Fields]) AND ("minoxidil"[MeSH Terms] OR "minoxidil"[All Fields]) |
| (((((androgenic alopecia) OR (male pattern baldness)) OR (female pattern baldness)) OR (hair loss)) AND (PRF)) AND (minoxidil) | ("alopecia"[MeSH Terms] OR "alopecia"[All Fields] OR ("androgenic"[All Fields] AND "alopecia"[All Fields]) OR "androgenic alopecia"[All Fields] OR ("alopecia"[MeSH Terms] OR "alopecia"[All Fields] OR ("male"[All Fields] AND "pattern"[All Fields] AND "baldness"[All Fields]) OR "male pattern baldness"[All Fields]) OR ("alopecia"[MeSH Terms] OR "alopecia"[All Fields] OR ("female"[All Fields] AND "pattern"[All Fields] AND "baldness"[All Fields]) OR "female pattern baldness"[All Fields]) OR ("alopecia"[MeSH Terms] OR "alopecia"[All Fields] OR ("hair"[All Fields] AND "loss"[All Fields]) OR "hair loss"[All Fields])) AND "PRF"[All Fields] AND ("minoxidil"[MeSH Terms] OR "minoxidil"[All Fields]) |
| (((((androgenic alopecia) OR (male pattern baldness)) OR (female pattern baldness)) OR (hair loss)) AND (platelet rich fibrin)) AND (minoxidil) | ("alopecia"[MeSH Terms] OR "alopecia"[All Fields] OR ("androgenic"[All Fields] AND "alopecia"[All Fields]) OR "androgenic alopecia"[All Fields] OR ("alopecia"[MeSH Terms] OR "alopecia"[All Fields] OR ("male"[All Fields] AND "pattern"[All Fields] AND "baldness"[All Fields]) OR "male pattern baldness"[All Fields]) OR ("alopecia"[MeSH Terms] OR "alopecia"[All Fields] OR ("female"[All Fields] AND "pattern"[All Fields] AND "baldness"[All Fields]) OR "female pattern baldness"[All Fields]) OR ("alopecia"[MeSH Terms] OR "alopecia"[All Fields] OR ("hair"[All Fields] AND "loss"[All Fields]) OR "hair loss"[All Fields])) AND ("platelet rich fibrin"[MeSH Terms] OR ("platelet rich"[All Fields] AND "fibrin"[All Fields]) OR "platelet rich fibrin"[All Fields] OR ("platelet"[All Fields] AND "rich"[All Fields] AND "fibrin"[All Fields]) OR "platelet rich fibrin"[All Fields]) AND ("minoxidil"[MeSH Terms] OR "minoxidil"[All Fields]) |
| (((((androgenic alopecia) OR (male pattern baldness)) OR (female pattern baldness)) OR (hair loss)) AND (platelet rich plasma)) AND (minoxidil)) | ("alopecia"[MeSH Terms] OR "alopecia"[All Fields] OR ("androgenic"[All Fields] AND "alopecia"[All Fields]) OR "androgenic alopecia"[All Fields] OR ("alopecia"[MeSH Terms] OR "alopecia"[All Fields] OR ("male"[All Fields] AND "pattern"[All Fields] AND "baldness"[All Fields]) OR "male pattern baldness"[All Fields]) OR ("alopecia"[MeSH Terms] OR "alopecia"[All Fields] OR ("female"[All Fields] AND "pattern"[All Fields] AND "baldness"[All Fields]) OR "female pattern baldness"[All Fields]) OR ("alopecia"[MeSH Terms] OR "alopecia"[All Fields] OR ("hair"[All Fields] AND "loss"[All Fields]) OR "hair loss"[All Fields])) AND ("platelet rich plasma"[MeSH Terms] OR ("platelet rich"[All Fields] AND "plasma"[All Fields]) OR "platelet rich plasma"[All Fields] OR ("platelet"[All Fields] AND "rich"[All Fields] AND "plasma"[All Fields]) OR "platelet rich plasma"[All Fields]) AND ("minoxidil"[MeSH Terms] OR "minoxidil"[All Fields]) |
