# Supplementary material for: The additive value of platelet-rich plasma to topical Minoxidil in the treatment of androgenetic alopecia: A systematic review and meta-analysis
Source: PLoS One. 2024 Aug 28;19(8):e0308986. doi: 10.1371/journal.pone.0308986 (PMC11356437; doi:10.1371/journal.pone.0308986)
Supplement: S3 Table — (DOCX) [file pone.0308986.s003.docx]

Supplementary Table 3: List of excluded studies

| Excluded study | Reason |
| --- | --- |
| Shah et al[1] | Non-RCT |
| Jha et al[2] | Non-RCT |
| Pakhomova et al[3] | Non-RCT |
| Dubin et al[4] | Not using PRP/PRF as add on therapy |
| Siah et al[5] | Not using PRP/PRF as add on therapy |
| Moftah et al[6] | Not using PRP/PRF as add on therapy |
| Trink et al[7] | Not using PRP/PRF as add on therapy |
| Bayat et al[8] | Not using PRP/PRF as add on therapy |
| Ghafoor et al[9] | Not using PRP/PRF as add on therapy |
| Hegde et al[10] | Not using PRP/PRF as add on therapy |
| Rinaldi et al[11] | Not using PRP/PRF as add on therapy |
| Wei et al[12] | Not using PRP/PRF as add on therapy |
| Balasundaram et al[13] | Not using PRP/PRF as add on therapy |
| Sasaki et al[14] | Not using PRP/PRF as add on therapy |
| Gentile et al[15] | Not using PRP/PRF as add on therapy |

References

1. Shah KB, Shah AN, Solanki RB, Raval RC. A Comparative Study of Microneedling with Platelet-rich Plasma Plus Topical Minoxidil (5%) and Topical Minoxidil (5%) Alone in Androgenetic Alopecia. Int J Trichology. 2017;9: 14–18. doi:10.4103/ijt.ijt_75_16

2. Jha AK, Zeeshan M, Singh A, Roy PK. Platelet-rich plasma with low dose oral minoxidil (1.25mg versus 2.5mg) along with trichoscopic pre- and post-treatment evaluation. J Cosmet Dermatol. 2021;20: 3220–3226. doi:10.1111/jocd.14049

3. Pakhomova EE, Smirnova IO. Comparative Evaluation of the Clinical Efficacy of PRP-Therapy, Minoxidil, and Their Combination with Immunohistochemical Study of the Dynamics of Cell Proliferation in the Treatment of Men with Androgenetic Alopecia. Int J Mol Sci. 2020;21. doi:10.3390/ijms21186516

4. Dubin DP, Lin MJ, Leight HM, Farberg AS, Torbeck RL, Burton WB, et al. The effect of platelet-rich plasma on female androgenetic alopecia: A randomized controlled trial. J Am Acad Dermatol. 2020;83: 1294–1297. doi:10.1016/j.jaad.2020.06.1021

5. Siah TW, Guo H, Chu T, Santos L, Nakamura H, Leung G, et al. Growth factor concentrations in platelet-rich plasma for androgenetic alopecia: An intra-subject, randomized, blinded, placebo-controlled, pilot study. Exp Dermatol. 2020;29: 334–340. doi:10.1111/exd.14074

6. Moftah NH, Taha NE-E, Alhabibi AM, Hamdino M. Different platelet-rich plasma preparation protocols in Female pattern hair loss: Does it affect the outcome? A pilot study. J Cosmet Dermatol. 2022;21: 3316–3326. doi:10.1111/jocd.14648

7. Trink A, Sorbellini E, Bezzola P, Rodella L, Rezzani R, Ramot Y, et al. A randomized, double-blind, placebo- and active-controlled, half-head study to evaluate the effects of platelet-rich plasma on alopecia areata. Br J Dermatol. 2013;169: 690–4. doi:10.1111/bjd.12397

8. Bayat M, Yazdanpanah MJ, Hamidi Alamdari D, Banihashemi M, Salehi M. The effect of platelet-rich plasma injection in the treatment of androgenetic alopecia. J Cosmet Dermatol. 2019;18: 1624–1628. doi:10.1111/jocd.12907

9. Ghafoor R, Saher N, Ali SM. The Role of 5% Minoxidil <em>versus</em> Platelet-Rich Plasma in Treatment of Alopecia Areata. J Coll Physicians Surg Pak. 2024;34: 650–653. doi:10.29271/jcpsp.2024.06.650

10. Hegde P, Relhan V, Sahoo B, Garg VK. A randomized, placebo and active controlled, split scalp study to evaluate the efficacy of platelet-rich plasma in patchy alopecia areata of the scalp. Dermatol Ther. 2020;33: e14388. doi:10.1111/dth.14388

11. Rinaldi F, Marzani B, Pinto D, Sorbellini E. Randomized controlled trial on a PRP-like cosmetic, biomimetic peptides based, for the treatment of alopecia areata. J Dermatolog Treat. 2019;30: 588–593. doi:10.1080/09546634.2018.1544405

12. Wei W, Zhang Y, Long B, Zhang Y, Zhang C, Zhang S. Injections of platelet-rich plasma prepared by automatic blood cell separator combined with topical 5% minoxidil in the treatment of male androgenetic alopecia. Skin Res Technol. 2023;29: e13315. doi:10.1111/srt.13315

13. Balasundaram M, Kumari R, Ramassamy S. Efficacy of autologous platelet-rich plasma therapy versus topical Minoxidil in men with moderate androgenetic alopecia: a randomized open-label trial. J Dermatolog Treat. 2023;34: 2182618. doi:10.1080/09546634.2023.2182618

14. Sasaki GH. The Effects of Lower vs Higher Cell Number of Platelet-Rich Plasma (PRP) on Hair Density and Diameter in Androgenetic Alopecia (AGA): A Randomized, Double-Blinded, Placebo, Parallel-Group Half-Scalp IRB-Approved Study. Aesthetic Surg J. 2021;41: NP1659–NP1672. doi:10.1093/asj/sjab236

15. Gentile P, Garcovich S. Autologous activated platelet-rich plasma (AA-PRP) and non-activated (A-PRP) in hair growth: a retrospective, blinded, randomized evaluation in androgenetic alopecia. Expert Opin Biol Ther. 2020;20: 327–337. doi:10.1080/14712598.2020.1724951
