# Supplementary material for: The additive value of platelet-rich plasma to topical Minoxidil in the treatment of androgenetic alopecia: A systematic review and meta-analysis
Source: PLoS One. 2024 Aug 28;19(8):e0308986. doi: 10.1371/journal.pone.0308986 (PMC11356437; doi:10.1371/journal.pone.0308986)
Supplement: S4 Table — (DOCX) [file pone.0308986.s004.docx]

Supplementary Table 4: Quantitative data extracted from studies

| Study | Follow-up | Hair density | | Sample size | Adverse drug reaction | |
| --- | --- | --- | --- | --- | --- | --- |
|  |  | Intervention | Control |  | Intervention | Control |
| Wu 2023 | 1  3  6 | 144.58± 18.85  153.18± 22  157.89± 19.86 | 153.96± 20.21  175.33± 24.52  182.89± 25.39 | 25  25 | 2 | 3 |
| Pachar 2022 | 6 | 113± 7.6 | 104.8± 4.97 | 50  50 | 4 | 5 |
| Gowda 2021 | 3  5 | 76.73+12.66  86.13+14.82 | 61.53 +13.53  66.83+16.73 | 30  30 | NR | NR |
| Ramadan 2021 |  | NR | NR | NR | NR | NR |
| Singh 2020 | 1  3  5 | 109.60±35.09  139.00±32.90  150.45±28.27 | 90.30±41.81  99.50±38.67  123.95±35.41 | 20  20 | 3 | 7 |

NR, not reported
