# Supplementary material for: The additive value of platelet-rich plasma to topical Minoxidil in the treatment of androgenetic alopecia: A systematic review and meta-analysis
Source: PLoS One. 2024 Aug 28;19(8):e0308986. doi: 10.1371/journal.pone.0308986 (PMC11356437; doi:10.1371/journal.pone.0308986)
Supplement: S5 Table — (DOCX) [file pone.0308986.s005.docx]

**Supplementary Table 2: GRADE assessment of evidence**

| **Certainty assessment** | | | | | | | **№ of patients** | | **Effect** | | **Certainty** | **Importance** |
| --- | --- | --- | --- | --- | --- | --- | --- | --- | --- | --- | --- | --- |
| **№ of studies** | **Study design** | **Risk of bias** | **Inconsistency** | **Indirectness** | **Imprecision** | **Other considerations** | **PRP plus minoxidil** | **Minoxidil** | **Relative (95% CI)** | **Absolute (95% CI)** |  |  |
| **Hair density - One month** | | | | | | | | | | | | |
| 2 | randomised trials | serious^a^ | not serious | not serious | serious^b^ | none | 45 | 45 | - | MD **11.07 higher** (1.2 higher to 20.94 higher) | ⨁⨁◯◯ Low |  |
| **Hair density - Three months** | | | | | | | | | | | | |
| 3 | randomised trials | very serious^c^ | serious^d^ | not serious | serious^b^ | none | 75 | 75 | - | MD **21.81 higher** (10.62 higher to 33 higher) | ⨁◯◯◯ Very low |  |
| **Hair density - Five/six months** | | | | | | | | | | | | |
| 4 | randomised trials | very serious^e^ | serious^d^ | not serious | serious^b^ | none | 125 | 125 | - | MD **17.8 higher** (7.91 higher to 27.69 higher) | ⨁◯◯◯ Very low |  |
| **Adverse events** | | | | | | | | | | | | |
| 3 | randomised trials | very serious^f^ | not serious | not serious | serious^b^ | none | 9/95 (9.5%) | 15/95 (15.8%) | **OR 0.55** (0.22 to 1.36) | **64 fewer per 1,000** (from 118 fewer to 45 more) | ⨁◯◯◯ Very low |  |

**CI:** confidence interval; **MD:** mean difference; **OR:** odds ratio

#### Explanations

a. High risk of bias in the trial of Singh et al

b. Broad 95% Confidence intervals

c. High risk of bias in Singh et al and Gowda et al

d. High heterogeneity in the meta-analysis

e. High risk of bias in Singh et al, Gowda et al and Pachar et al

f. High risk of bias in Singh et al and Pachar et al
